# *N* ^1^,*N* ^2^-Bis(2-pyrid­yl)formamidine

**DOI:** 10.1107/S1600536809004851

**Published:** 2009-02-18

**Authors:** Chia-Jun Wu, Chang-Wei Su, Chun-Wei Yeh, Jhy-Der Chen, Ju-Chun Wang

**Affiliations:** a Department of Chemistry, Chung-Yuan Christian University, Chung-Li, Taiwan; b Department of Chemistry, Soochow University, Taipei, Taiwan

## Abstract

In the crystal structure of the title compound, C_11_H_10_N_4_, the dihedral angle between the two pyridyl rings is 36.1 (1)°. The mol­ecules are connected *via* two strong N—H⋯N and two weak C—H⋯N hydrogen bonds into dimers, which are located on centers of inversion. This compound adopts the *s–trans–anti–s–cis* conformation in the solid state.

## Related literature

For similar structures, see: Liang *et al.* (2003 ▶); Yang *et al.* (2000 ▶); Radak *et al.* (2001 ▶); Cotton *et al.* (1998 ▶). For the synthesis, see: Roberts (1949 ▶).
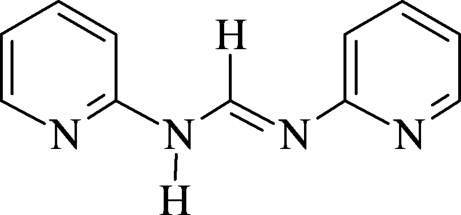


## Experimental

### 

#### Crystal data

C_11_H_10_N_4_
*M* *_r_* = 198.23
Monoclinic, 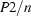

*a* = 11.0411 (14) Å
*b* = 4.3904 (5) Å
*c* = 20.789 (3) Å
β = 98.725 (2)°
*V* = 996.1 (2) Å^3^
*Z* = 4
Mo *K*α radiation
μ = 0.09 mm^−1^
*T* = 298 K
0.44 × 0.12 × 0.08 mm

#### Data collection

Bruker SMART CCD area-detector diffractometer
Absorption correction: empirical (using intensity measurements) (**SADABS**; Bruker, 1997 ▶) *T*_min_ = 0.983, *T*_max_ = 0.995
3628 measured reflections
1697 independent reflections
1251 reflections with *I* > 2σ(*I*)
*R*_int_ = 0.041

#### Refinement

*R*[*F* ^2^ > 2σ(*F* ^2^)] = 0.059
*wR*(*F* ^2^) = 0.143
*S* = 1.13
1697 reflections
141 parameters
H atoms treated by a mixture of independent and constrained refinement
Δρ_max_ = 0.14 e Å^−3^
Δρ_min_ = −0.19 e Å^−3^

### 

Data collection: *SMART* (Bruker, 1997 ▶); cell refinement: *SAINT* (Bruker, 1997 ▶); data reduction: *SAINT* and *SHELXTL* (Sheldrick, 2008 ▶); program(s) used to solve structure: *SHELXS97* (Sheldrick, 2008 ▶); program(s) used to refine structure: *SHELXL97* (Sheldrick, 2008 ▶); molecular graphics: *SHELXTL*; software used to prepare material for publication: *SHELXTL*.

## Supplementary Material

Crystal structure: contains datablocks I, global. DOI: 10.1107/S1600536809004851/nc2134sup1.cif

Structure factors: contains datablocks I. DOI: 10.1107/S1600536809004851/nc2134Isup2.hkl

Additional supplementary materials:  crystallographic information; 3D view; checkCIF report

## Figures and Tables

**Table 1:** Hydrogen-bond geometry (Å, °)

*D*—H⋯*A*	*D*—H	H⋯*A*	*D*⋯*A*	*D*—H⋯*A*
N3—H3N⋯N2^i^	0.90 (3)	2.14 (3)	3.044 (3)	175 (2)
C8—H8*A*⋯N1^i^	0.93	2.51	3.388 (4)	157

Symmetry code: (i) 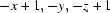.

**Fig. 1.**
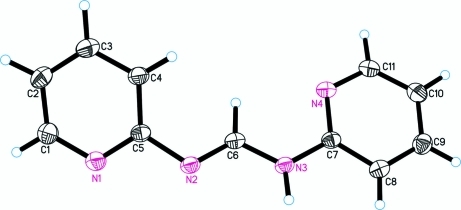
: An ORTEP diagram showing the structure of the title compound with labeling and displacement ellipsoids drawn at the 30 % probability level.

**Fig. 2.**
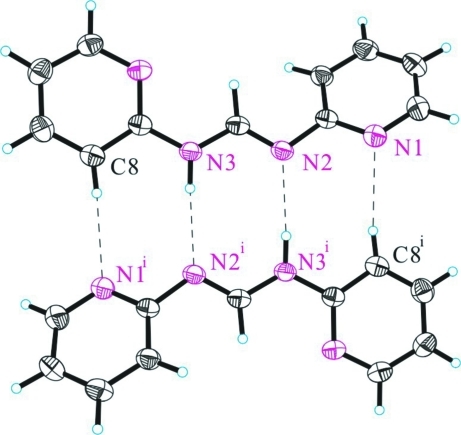
: View onto the dimers formed by intermolecular hydrogen bonding, which is shown as dashed lines. Symmetry code: (i) -x+1, -y, -z+1.

## supplementary crystallographic information

### Comment

The title compound and its anion have been used as bridging ligands in coordination chemistry (Liang *et al.*, 2003; Yang *et al.*, 2000; Radak *et al.*, 2001; Cotton *et al.*, 1998). In the present work, the structure of the title compound (Fig. 1) has been determined to explore its ligand conformation. In the crystal structure of the title compound the molecule is in a *s-trans-anti-s-cis *conformation. This conformation is different from that in the Re complex, which is *s-cis-syn-s-cis*(Liang *et al.*, 2003).

Thus, the conformation of the free ligand has been changed upon coordination to the metal center. The molecules are connected via two strong N—H—N and two weak C—H—N hydrogen bonds into dimers, which are located on centres of inversion (Fig. 2),

### Experimental

The title compound was prepared according to a published procedure (Roberts, 1949). 2-Aminopyridine (11.28 g, 0.12 mol) and triethyl orthoformate (8.88 g, 0.06 mol) were placed in a flask under nitrogen. The mixture was then refluxed for 8 h to give a brown solid. Dichloromethane was then added to dissolve the solid and then hexanes added to induce the precipitate. The precipitate was filtered and dried under vacuum to give a light yellow solid with a yield of 82 %. Crystals suitable for X-ray crystallography were obtained by dissolving the product in dichloromethane, followed by slow evaporation of the solvent.

### Refinement

Pyridyl and methine H atoms were positioned with ideal geometry and were refined isotropic with C—H = 0.93 Å and *U*_iso_(H) = 1.2U_eq_(C) using a riding model. The amine H atom was found in fourier difference map and refined isotropically.

### Crystal data

**Table:**

C_11_H_10_N_4_	*F*(000) = 416
*M**_r_* = 198.23	*D*_x_ = 1.322 Mg m^−^^3^
Monoclinic, *P*2/*n*	Mo *K*α radiation, λ = 0.71073 Å
*a* = 11.0411 (14) Å	Cell parameters from 1493 reflections
*b* = 4.3904 (5) Å	θ = 2.0–25.1°
*c* = 20.789 (3) Å	µ = 0.09 mm^−^^1^
β = 98.725 (2)°	*T* = 298 K
*V* = 996.1 (2) Å^3^	Column, colorless
*Z* = 4	0.44 × 0.12 × 0.08 mm

### Data collection

**Table:**

Bruker SMART CCD area-detector diffractometer	1697 independent reflections
Radiation source: fine-focus sealed tube	1251 reflections with *I* > 2σ(*I*)
graphite	*R*_int_ = 0.041
φ and ω scans	θ_max_ = 25.1°, θ_min_ = 2.0°
Absorption correction: empirical (using intensity measurements) (*SADABS*; Bruker, 1997)	*h* = −13→8
*T*_min_ = 0.983, *T*_max_ = 0.995	*k* = −5→4
3628 measured reflections	*l* = −23→24

### Refinement

**Table:**

Refinement on *F*^2^	Secondary atom site location: difference Fourier map
Least-squares matrix: full	Hydrogen site location: inferred from neighbouring sites
*R*[*F*^2^ > 2σ(*F*^2^)] = 0.059	H atoms treated by a mixture of independent and constrained refinement
*wR*(*F*^2^) = 0.143	*w* = 1/[σ^2^(*F*_o_^2^) + (0.0509*P*)^2^ + 0.3516*P*] where *P* = (*F*_o_^2^ + 2*F*_c_^2^)/3
*S* = 1.13	(Δ/σ)_max_ < 0.001
1697 reflections	Δρ_max_ = 0.14 e Å^−^^3^
141 parameters	Δρ_min_ = −0.19 e Å^−^^3^
0 restraints	Extinction correction: *SHELXL97* (Sheldrick, 2008), Fc^*^=kFc[1+0.001xFc^2^λ^3^/sin(2θ)]^-1/4^
Primary atom site location: structure-invariant direct methods	Extinction coefficient: 0.024 (4)

### Special details

**Table:**

Geometry. All esds (except the esd in the dihedral angle between two l.s. planes) are estimated using the full covariance matrix. The cell esds are taken into account individually in the estimation of esds in distances, angles and torsion angles; correlations between esds in cell parameters are only used when they are defined by crystal symmetry. An approximate (isotropic) treatment of cell esds is used for estimating esds involving l.s. planes.
Refinement. Refinement of F^2^ against ALL reflections. The weighted R-factor wR and goodness of fit S are based on F^2^, conventional R-factors R are based on F, with F set to zero for negative F^2^. The threshold expression of F^2^ > 2sigma(F^2^) is used only for calculating R-factors(gt) etc. and is not relevant to the choice of reflections for refinement. R-factors based on F^2^ are statistically about twice as large as those based on F, and R- factors based on ALL data will be even larger.

### Fractional atomic coordinates and isotropic or equivalent isotropic displacement parameters (Å^2^)

**Table:**

	*x*	*y*	*z*	*U*_iso_*/*U*_eq_	
N1	0.7611 (2)	−0.1912 (6)	0.64055 (11)	0.0633 (7)	
N2	0.66575 (17)	−0.0267 (5)	0.53928 (10)	0.0502 (6)	
N3	0.58806 (19)	0.2810 (5)	0.45290 (10)	0.0482 (6)	
N4	0.71668 (18)	0.5763 (5)	0.39863 (10)	0.0536 (6)	
C1	0.8583 (3)	−0.3057 (8)	0.67908 (14)	0.0731 (9)	
H1B	0.8510	−0.3453	0.7223	0.088*	
C2	0.9685 (3)	−0.3685 (8)	0.65886 (15)	0.0708 (9)	
H2B	1.0342	−0.4453	0.6876	0.085*	
C3	0.9785 (3)	−0.3143 (7)	0.59503 (15)	0.0675 (8)	
H3A	1.0516	−0.3553	0.5796	0.081*	
C4	0.8802 (2)	−0.1991 (7)	0.55388 (13)	0.0559 (7)	
H4B	0.8856	−0.1632	0.5103	0.067*	
C5	0.7723 (2)	−0.1370 (6)	0.57845 (12)	0.0483 (6)	
C6	0.6813 (2)	0.1693 (6)	0.49522 (12)	0.0480 (6)	
H6A	0.7604	0.2361	0.4926	0.058*	
C7	0.6029 (2)	0.4915 (6)	0.40425 (12)	0.0471 (6)	
C8	0.5000 (2)	0.6016 (6)	0.36432 (13)	0.0558 (7)	
H8A	0.4219	0.5384	0.3699	0.067*	
C9	0.5161 (3)	0.8048 (7)	0.31657 (14)	0.0632 (8)	
H9A	0.4487	0.8817	0.2891	0.076*	
C10	0.6329 (3)	0.8952 (7)	0.30926 (14)	0.0636 (8)	
H10A	0.6462	1.0314	0.2768	0.076*	
C11	0.7286 (2)	0.7769 (7)	0.35157 (13)	0.0596 (8)	
H11A	0.8073	0.8405	0.3473	0.072*	
H3N	0.512 (3)	0.203 (6)	0.4525 (12)	0.058 (8)*	

### Atomic displacement parameters (Å^2^)

**Table:**

	*U*^11^	*U*^22^	*U*^33^	*U*^12^	*U*^13^	*U*^23^
N1	0.0500 (13)	0.0826 (18)	0.0570 (14)	−0.0025 (12)	0.0077 (11)	0.0050 (13)
N2	0.0373 (11)	0.0612 (14)	0.0520 (12)	−0.0022 (10)	0.0069 (9)	−0.0015 (11)
N3	0.0370 (11)	0.0514 (13)	0.0562 (13)	−0.0033 (10)	0.0070 (10)	0.0006 (11)
N4	0.0438 (12)	0.0592 (14)	0.0597 (13)	−0.0022 (10)	0.0144 (10)	−0.0004 (11)
C1	0.0608 (18)	0.097 (2)	0.0590 (17)	0.0002 (18)	0.0027 (14)	0.0105 (17)
C2	0.0492 (16)	0.084 (2)	0.075 (2)	0.0047 (16)	−0.0052 (14)	0.0032 (18)
C3	0.0455 (15)	0.079 (2)	0.079 (2)	0.0036 (15)	0.0121 (14)	−0.0029 (18)
C4	0.0445 (14)	0.0701 (19)	0.0538 (15)	0.0014 (13)	0.0100 (12)	−0.0024 (14)
C5	0.0407 (13)	0.0499 (15)	0.0541 (15)	−0.0058 (11)	0.0061 (11)	−0.0039 (12)
C6	0.0383 (13)	0.0518 (15)	0.0547 (14)	−0.0026 (12)	0.0100 (11)	−0.0094 (13)
C7	0.0435 (14)	0.0472 (14)	0.0515 (14)	0.0005 (12)	0.0106 (11)	−0.0090 (13)
C8	0.0458 (15)	0.0579 (17)	0.0631 (16)	0.0007 (13)	0.0067 (12)	−0.0030 (14)
C9	0.0622 (18)	0.0610 (18)	0.0649 (18)	0.0097 (15)	0.0051 (14)	0.0005 (15)
C10	0.0713 (19)	0.0610 (19)	0.0617 (17)	0.0038 (15)	0.0207 (15)	0.0031 (15)
C11	0.0535 (16)	0.0632 (19)	0.0660 (18)	−0.0029 (14)	0.0214 (14)	−0.0008 (15)

### Geometric parameters (Å, °)

**Table:**

N1—C1	1.337 (4)	C3—C4	1.372 (4)
N1—C5	1.337 (3)	C3—H3A	0.9300
N2—C6	1.287 (3)	C4—C5	1.392 (3)
N2—C5	1.411 (3)	C4—H4B	0.9300
N3—C6	1.342 (3)	C6—H6A	0.9300
N3—C7	1.398 (3)	C7—C8	1.388 (3)
N3—H3N	0.90 (3)	C8—C9	1.366 (4)
N4—C7	1.332 (3)	C8—H8A	0.9300
N4—C11	1.337 (3)	C9—C10	1.380 (4)
C1—C2	1.374 (4)	C9—H9A	0.9300
C1—H1B	0.9300	C10—C11	1.370 (4)
C2—C3	1.369 (4)	C10—H10A	0.9300
C2—H2B	0.9300	C11—H11A	0.9300
			
C1—N1—C5	117.5 (2)	C4—C5—N2	122.7 (2)
C6—N2—C5	116.7 (2)	N2—C6—N3	122.6 (2)
C6—N3—C7	123.6 (2)	N2—C6—H6A	118.7
C6—N3—H3N	118.9 (17)	N3—C6—H6A	118.7
C7—N3—H3N	117.1 (17)	N4—C7—C8	123.2 (3)
C7—N4—C11	116.5 (2)	N4—C7—N3	117.6 (2)
N1—C1—C2	124.1 (3)	C8—C7—N3	119.2 (2)
N1—C1—H1B	118.0	C9—C8—C7	118.5 (3)
C2—C1—H1B	118.0	C9—C8—H8A	120.8
C3—C2—C1	117.8 (3)	C7—C8—H8A	120.8
C3—C2—H2B	121.1	C8—C9—C10	119.6 (3)
C1—C2—H2B	121.1	C8—C9—H9A	120.2
C2—C3—C4	119.7 (3)	C10—C9—H9A	120.2
C2—C3—H3A	120.1	C11—C10—C9	117.6 (3)
C4—C3—H3A	120.1	C11—C10—H10A	121.2
C3—C4—C5	118.9 (3)	C9—C10—H10A	121.2
C3—C4—H4B	120.6	N4—C11—C10	124.6 (3)
C5—C4—H4B	120.6	N4—C11—H11A	117.7
N1—C5—C4	122.0 (2)	C10—C11—H11A	117.7
N1—C5—N2	115.2 (2)		

### Hydrogen-bond geometry (Å, °)

**Table:**

*D*—H···*A*	*D*—H	H···*A*	*D*···*A*	*D*—H···*A*
N3—H3N···N2^i^	0.90 (3)	2.14 (3)	3.044 (3)	175 (2)
C8—H8A···N1^i^	0.93	2.51	3.388 (4)	157

Symmetry codes: (i) −*x*+1, −*y*, −*z*+1.
